# Enzymic determination of glucose with SMAC: adaption of the dichlorophenol/ aminophenazone chromogen

**DOI:** 10.1155/S1463924681000436

**Published:** 1981

**Authors:** F. J. Gella, T. Olivella, J. Gener, R. Galimany, M. J. Castiñeiras

**Affiliations:** 1 Department of Research and Development Knickerbocker Laboratories Borrell 158 Barcelona–15 Spain; 2 Seccion de Automatizacion Departamento de Análisis Clinicos Residencia Sanitaria “Principes de España” Hospitalet de Llobregat (Barcelona) Spain


### Enzymic determination of glucose with SMAC: adaption of the dichlorophenol/aminophenazone chromogen

**F. J. Gella, T. Olivella and J. Gener**
*Department of Research and Development, Knickerbocker Laboratories, Borrell 158, Barcelona – 15, Spain.*

**R. Galimany and M.J. Castiñeiras**
*Seccion de Automatizacion, Departamento de Análisis Clinicos, Residencia Sanitaria "Principes de España", Hospitalet de Llobregat (Barcelona), Spain.*

### Introduction

The currently used SMAC glucose oxidase method is a modification of the procedure of Gochman and Schmitz [1]. In this colorimetirc determination, the specificity of glucose oxidase is combined with a peroxidase indicator coupler, 3-methyl-2 benzothiazolinone hydrazone (MBTH) and dimethylaniline (DMA) to form a coloured indamine dye with a high molar absorption. However, as much as six different reagents have to be prepared by the user, some of them daily. Barham and Trinder [2] described in 1972 a method involving the use of 4-aminophenazone as a colour coupler with sulphonated 2,4-dichlorophenol for determining the hydrogen peroxide produced from glucose with glucose oxidase. This method had a high sensitivity and the additional possibility of using a single reagent stable for several days. This paper presents an adaptation of the former to the SMAC system considerably reducing both the number of reagents involved and the operator-time consumed. The proposed method was compared with the hexokinase manual method and the Gochman-Schmitz method for the SMAC.

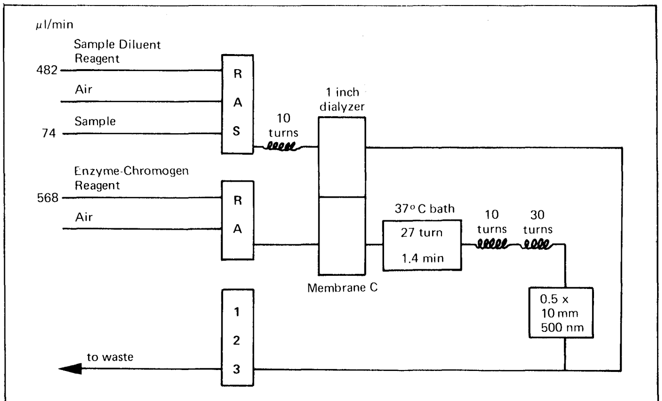

*Figure 1. Simplified flow diagram for the glucose channel (DCPS/AP system).*

### Materials and methods

### Reagents

*Reagents for SMAC glucose determination (MBTH/DMA)*
Reagents were prepared according to the manufacturer [3].
*Glucose oxidase working reagent:* 1.6 ml of glucose oxidase stock reagent (1000 U/l) were diluted with 163 ml of phosphate buffer 0.1 mol/l, pH 7.0 and 0.16 ml of 300 g/l Brij-35.
*MBTH/DMA working reagent:* 2.0 ml of 1 g/l MBTH and 3.0 ml of 10 ml/l DMA were diluted with 95.0 ml of 0.5 mol/l acetic acid. DMA and MBTH solutions were prepared in 0.1 mol/l hydrochloric acid.
*Peroxidase:* 19 U/ml in distilled water.
*Glucose sample diluent:* 9 g/l saline-0.3 g/l Brij-35.

*Reagents proposed for SMAC glucose determination (DCPS/AP)*
*Enzyme/chromogen mixture:* The reagent contains, per litre: 0.8 mmol aminophenazone, 10 mmol sulphonated

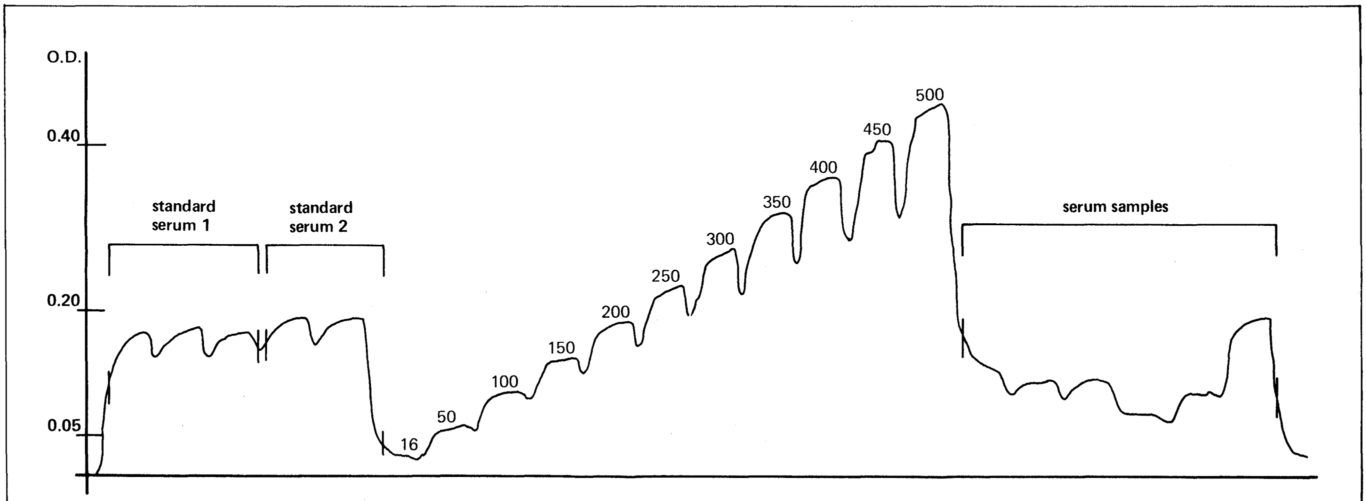

*Figure 2. Representative recording of glucose determinations. Number above the peaks indicate the concentration of glucose (mg/100 ml).*

2,4-dichlorophenol, 10000 U glucose oxidase and 1100 U peroxidase in phosphate buffer 0.1 mol/l, pH 7.4. It can be prepared in a stable powdered or lyophilised form suitable for up to four days use when appropriately dissolved in water. *Glucose sample diluent:* 0.3 g/l Brij-35.

### Instrumentation

The instrument used was a SMAC (Technicon Instruments Corp, Tarrytown, NY, 10591). The flow diagram for the DCPS/AP proposed method is shown in Figure 1. Modifications of the original channel are minimal: substitution of the interference filter (500 nm for 600 nm) and disconnection of the MBTH/DMA and peroxidase tubing.

### Results and discussion

### Linearity

Readings and glucose concentrations were linearly related up to 27.5 mmol/l. A recording of the procedure for continuous sampling of standard sera, an ascending standard curve and results with representative sera are reproduced in Figure 2.

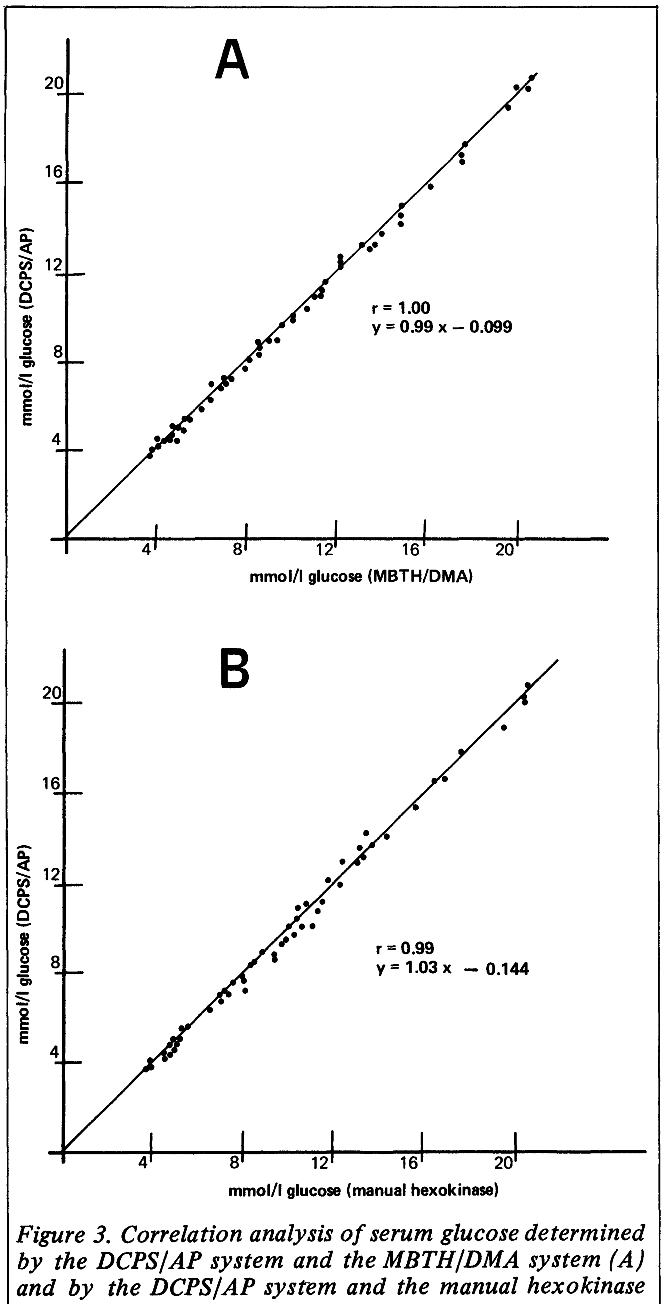

*Figure 3. Correlation analysis of serum glucose determined by the DCPS/AP system and the MBTH/DMA system (A) and by the DCPS/AP system and the manual hexokinase procedure (B).*

**Table 1. Day-to-day precision of automated glucose method (10 daily analysis).**

| | Serum A | | Serum B | |
|---|---|---|---|---|
| | MBTH/DMA system | DCPS/AP system | MBTH/DMA system | DCPS/AP system |
| Mean (mmol/l) | 4.17 | 4.21 | 13.37 | 13.57 |
| SD | 0.15 | 0.12 | 0.33 | 0.28 |
| CV(%) | 3.60 | 2.85 | 2.47 | 2.06 |
| n | 25 | 25 | 25 | 25 |

**Table 2. Interferences study. Solutions of the examined compounds were added to a serum pool and the glucose concentration was tested and compared with the same pool diluted with saline. Glucose found is the mean of triplicate results.**

| Added Substance | Concentration tested (mg/ml) | Normal concentration (mg/ml)* | Glucose found (mmol/l) |
|---|---|---|---|
| Saline | – | – | 6.39 |
| Heparine | 150,000 U/l | – | 6.39 |
| Citrate | 200 | – | 6.33 |
| EDTA | 60 | – | 6.50 |
| NaF | 300 | – | 6.39 |
| Oxalate | 30 | – | 6.44 |
| Glutathione | 30 | 3.1 | 5.83 |
| | 7 | 3.1 | 6.44 |
| Ascorbic acid | 1 | 0.3 | 6.33 |
| | 5 | 0.3 | 4.67 |
| Uric acid | 1 | 0.5 | 6.39 |
| Bilirubin | 2 | 0.1 | 6.44 |
| Cysteine | 7.5 | 0.001 | 6.33 |
| Fructose | 30 | – | 6.39 |
| Mannose | 30 | – | 6.39 |
| Xylose | 30 | – | 6.33 |
| Maltose | 30 | – | 23.33 |
| Galactose | 30 | – | 6.39 |
| Saccharose | 30 | – | 6.44 |
| Dextran | 30 | – | 6.36 |
| Creatinine | 2 | 0.1 | 6.39 |
| Salicylic acid | 2 | – | 6.39 |
| Acetylsalicylic acid | 10 | – | 6.33 |
| Gentisic acid | 0.2 | 0.04 | 6.39 |
| L-DOPA | 0.1 | 0.03-0.003 | 6.39 |
| Haemoglobin | 50 | – | 6.44 |
| Haemolysate | 50 | – | 4.72 |

**Approximate normal values of the compound in serum (physiological or therapeutical).*

### Precision

The precision of the method was determined by daily analysis of 2 lyophilised control sera with low and high glucose values. The results are shown in Table 1. Data shows satisfactory precision for consecutive runs involving change of reagents, standards, etc.

### Interferences

Various anticoagulants, drugs and metabolites were tested for potential interferences with the method (Table 2). A number of saccharides did not interfere reflecting the specificity of the method, maltose being an exception. The interference from maltose was due to the common contamination of glucose oxidase with maltase. None of the anticoagulants tested interfered. Glutathione, uric acid, ascorbic acid and bilirubin did not interfere at concentrations

**Table 3. Recovery of glucose added to dialysed serum. Found glucose is average of triplicate determinations.**

| Added glucose (mmol/l) | Found glucose (mmol/l) | Recovery (%) |
|---|---|---|
| 2.78 | 2.83 | 102 |
| 5.56 | 5.50 | 99 |
| 13.89 | 14.17 | 102 |
| 22.22 | 22.44 | 101 |

higher than those found in serum. The serum drug concentrations studied here were higher than would be expected after therapeutic doses and no interference was observed. Haemoglobin did not interfere up to 50 mg/ml, but a haemolysate with the same concentration of haemoglobin produced a decrease in the observed glucose value.

**Accuracy and comparison of methods**

Accuracy of the method was investigated by recovery studies and by comparison with the spectrophotometric hexokinase method. To investigate recovery a pool of human serum was prepared by dialysing it overnight against saline. Various amounts of glucose solution (555 mmol/l) and water were added to the dialysed serum and analysed. The results are shown in Table 3. Recovery was excellent at low and high glucose concentration. We compared our results for this method to those obtained by the Boehringer-Mannheim hexokinase manual procedure and by the MBTH/DMA SMAC method for 85 freshly collected patients' sera. The data were correlated (Figure 3) and showed excellent agreement over a wide range of plasma glucose values.

In conclusion, the proposed system is simpler and more economical than the MBTH/DMA method, performing similarly in linearity, sensitivity, resistance to interfering substances, accuracy and precision.

Recently obtained data have shown a similar performance of the proposed method in the SMA II system modified in the glucose channel as described for the SMAC (results not shown).

REFERENCES

[1] Gochman, N., and Schmitz, J. M., (1972), Application of a new peroxide indicator reaction to the specific, automated determination of glucose with glucose oxidase, *Clinical Chemistry*, **18**, 943-950.

[2] Barham, D., and Trinder, P., (1972), An improved colour reagent for the determination of blood glucose by the oxidase system, *Analyst*, **97**, 142-145.

[3] Technicon method no. SG4-0002PM4, (1974), Glucose, Technicon Instruments Corporation, Tarrytown, NY, 10591.

[4] Ibbott, F. A., (1974), Amino acids and related substances. In "Clinical Chemistry: Principles and Technics", 2 nd ed, R. J. Henry, D. C. Cannon and J. W. Winkelman, Eds. Harper and Row, Hagerstown, Md, p. 618.

[5] Schzauzer, G. N. and Rhead, W. J., (1973), Ascorbic acid abuse: Effects of long ingestion of excessive amounts on blood levels an urinary excretion, *International Journal of Vitamins and Nutritional Research*, **43**, 201.

[6] Roseman, S., and Dorfman, A., (1951), The determination and metabolism of gentisic acid, *Journal of Biological Chemistry*, **192**, 105.

[7] Muenter, M. D., and Tyce, G. M., (1971), L-DOPA therapy of Parkinson's disease: Plasma L-DOPA concentration, Therapeutic response, and side effects, *Mayo Clinical Proceedings*, **46**, 231.

[8] Hare, T. A., Vanna, S., Beasley, B., Chambers, R., and Vogel, W. H., (1971), Amino acid and DOPA levels in plasma and urine from L-DOPA treated patients with Parkinson's disease, *Journal of Laboratory and Clinical Medicine*, **77**, 319-325.

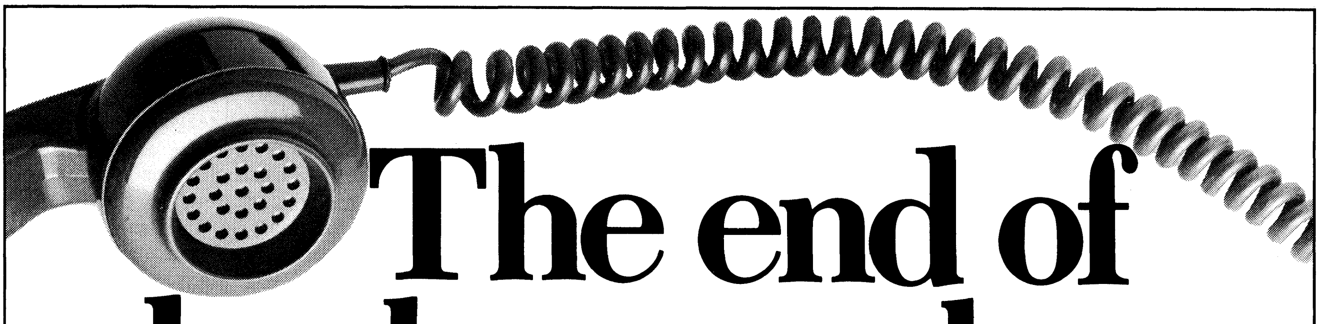

**The end of a broken analyser.**

A telephone is all it takes to keep your autoanalysis equipment in good shape.

One call to Sterilin Instruments brings you everything you could possibly need. Quickly, and economically.

From emergency repairs to preventative maintenance.

And from custom built manifolds to a vast range of accessories such as tubing, coils, stream dividers, dialyser plates, reagent filters, and many many more.

All made to meet or surpass OEM specifications.

And all at prices that won't shatter your budget.

We've been busy looking after continuous flow analysers since 1972 and we know them inside-out.

Which means we can completely refurbish your existing equipment. Or supply a new system.

And we also stock a wide range of modules and spare parts.

Make a start by asking for our catalogue–we're just at the other end of your telephone.

**Sterilin Instruments**

You're safe with Sterilin

Sterilin Instruments Ltd., Mill Lane Estate, Alton, Hants GU34 2PL. Tel: Alton (0420) 82604/83052 Telex: 8812919 STERI G.

